# An independent component analysis reveals brain structural networks related to TNF-α in drug-naïve, first-episode major depressive disorder: a source-based morphometric study

**DOI:** 10.1038/s41398-020-00873-8

**Published:** 2020-06-10

**Authors:** Shingo Kakeda, Keita Watanabe, Hoa Nguyen, Asuka Katsuki, Koichiro Sugimoto, Natsuki Igata, Osamu Abe, Reiji Yoshimura, Yukunori Korogi

**Affiliations:** 1 Department of Diagnostic Radiology, Hirosaki University Graduate School of Medicine Radiology, Aomori, Japan; 2 Department of Radiology, University of Occupational and Environmental Health, Fukuoka, Japan; 3 Department of Psychiatry, University of Occupational and Environmental Health, Fukuoka, Japan; 4 Department of Radiology, Graduate School of Medicine, The University of Tokyo, Tokyo, Japan

**Keywords:** Human behaviour, Depression

## Abstract

In a previous mouse study, social defeat stress-induced microglial activation released tumor necrosis factor-α (TNF-α), leading to neuronal changes in the prefrontal cortex (PFC) and behavioral changes (anxiety). We aimed to investigate the relationship between gray-matter (GM) structural networks and serum TNF-α in patients with major depression disorder (MDD) using multivariate source-based morphometry (SBM). Forty-five first-episode and drug-naïve MDD patients and 38 healthy subjects (HSs) were recruited. High-resolution T1-weighted imaging was performed and serum TNF-α levels were measured in all MDD patients and HSs. After acquiring GM structural networks using SBM, we compared the Z-transformed loading coefficients (*Z*-scores) between MDD patients and HSs, and investigated the relationship between the *Z*-scores and the serum TNF-α levels in MDD patients. The serum TNF-α levels in MDD patients were significantly higher than those in HSs. We extracted two independent GM structural networks (the prefrontal network and the insula-temporal network) with significant differences between MDD patients and HSs (−0.305 ± 0.85 and 0.253 ± 0.82; *P* = 0.03 in the prefrontal network, and −0.268 ± 0.86 and 0.467 ± 0.71; *P* < 0.01 in the insula-temporal network). The serum TNF-α levels were significantly correlated with the *Z*-scores in the prefrontal network after Bonferroni correction (*r* = −0.419, *p* < 0.01); however, the correlation in the insula-temporal network was not significant (*r* = −0.290, *p* = 0.11). Elevated serum TNF-α levels in the early stage of MDD were associated with alteration of the prefrontal network.

## Introduction

Accumulating evidence suggests a role of inflammation in the pathogenesis of major depressive disorder (MDD)^1–3^. Tumor necrosis factor-α (TNF-α) is a proinflammatory cytokine that is largely produced by macrophages^4^ and which has been shown to have both neurotoxic and neuroprotective effects^5^. Pathophysiologically, elevated serum TNF-α may induce decreased neuronal synaptic plasticity, reduced neurotrophic factors, and reduced neurogenesis^6^. MDD patients have been reported to have increased serum TNF-α levels, and antidepressant treatment can reduce the TNF-α level^7,8^. Furthermore, anti-TNF-α therapy can help relieve depressive symptoms and repair cognitive impairment^9^. This evidence suggests that TNF-α may affect the brain morphology in MDD patients through processes related to neurodegeneration. A recent study showed that stress-induced microglial activation in mice released IL-1α and TNF-α in the prefrontal cortex (PFC), leading to atrophy of the PFC neurons and behavioral changes (social avoidance and anxiety)^10^. The TNF-α-induced neuronal changes in the PFC may therefore play a pathophysiological role in MDD.

To our knowledge, only one previous study has evaluated the relationship between brain morphology and the serum TNF-α levels in MDD patients^11^; however, the authors failed to find any relationship between them on voxel-based morphometry (VBM), which automatically segments brain images into voxel-wise measures of gray-matter (GM)^12^. The VBM approach is a univariate approach. In contrast, the recently introduced technique of source-based morphometry (SBM) is a multivariate approach, which provides a way to pool information across different voxels as well as identify unpredicted patterns^13^. SBM applies an independent component analysis (ICA) to a segmented image, arranges voxels into sets that contain similar information^13^, and acquires common morphological features of the GM concentration among individuals at the network level. Thus, this method is suitable for identifying novel networks.

In this study, to test the hypothesis that serum TNF-α might be associated with brain networks in the prefrontal area of MDD patients, we acquired GM structural networks using SBM. In addition, we recruited first-episode and drug-naïve MDD patients because antidepressant treatment affects brain morphometry^14^. Our purpose was to evaluate the differences in the GM structural networks of MDD patients and healthy subjects (HSs), and to investigate the relationship between the GM structural networks and the serum TNF-α levels in MDD patients.

## Materials and methods

### Participants

Human experiments were carried out in accordance with guidelines provided and approved by the Institutional Review Board of the University of Occupational and Environmental Health School of Medicine, Japan (approval number: H25-13). All of the participants provided their written informed consent to participate in the study.

In the current study, first-episode and drug-naïve patients with MDD were recruited. A psychiatrist (A.K., with 14 years of experience in psychiatry) diagnosed patients with MDD using a fully Structured Clinical Interview for the Diagnostic and Statistical Manual for Mental Disorders, Fourth Edition, Text revision (DSM-IV-TR) Research Version, Non-Patient Edition (SCID-I/NP). To qualify for the study, the patients with MDD must not have previously met the criteria for any DSM-IV-TR Axis I disorder based on interviews performed by a psychiatrist. In short, none of the MDD patients in this study had any past episodes of mood disorder. Furthermore, patients with mild cognitive impairment were excluded, mainly based on information about their activities of daily living from family members or caregivers. In addition, a brief cognitive examination including a serial 7s test and an assessment of the patient’s short-term memory was performed by an experienced psychiatrist.

The severity of depression was evaluated using the 17-item Hamilton Rating Scale for Depression (HAMD-17). Only patients with a total HAMD-17 score of ≥14 were eligible for inclusion in the study. Between March 2009 and June 2018, 57 consecutive patients with first-episode and drug-naïve MDD were recruited. From this initial sample, the psychiatrist excluded patients who met the following criteria: (a) a history of cognitive impairment, neurological disease, or the presence of either Axis I (schizophrenia, other affective disorders, etc.) or Axis II (personality disorders, mental retardation, etc.) psychiatric disorders (*n* = 6); (b) the presence of comorbid substance use disorders (*n* = 3); (c) unwillingness to provide informed consent (*n* = 2); (d) brain abnormalities (a brain tumor) on conventional magnetic resonance imaging (MRI) data (including T2-weighted images) (*n* = 1). Thus, a total of 45 right-handed, first-episode, drug-naïve patients with MDD were included (Table 1). Forty of the 45 patients had participated in our previously published studies^11^, which analyzed the brain volume and inflammation in MDD.

**Table 1:** Demographic characteristics, brain volumes, and values of TNF-α of participants.

	Healthy subjects	MDD patients	
	(*n* = 38)	(*n* = 45)	*p*-value
Age, years; mean, (range, SD)	43.1 (22–65, 11.3)	47.2 (20–73, 14.3)	0.15
Female, numbers	12	22	0.12
Body mass index		21.1 (3.1)	
Education years, mean (SD)		13.2 (2.3)	
Smokers (%)		18 (40)	
HAMD-17, mean of total scores (SD)		22.6 (5.9)	
Inpatients (%)		30 (67)	
History of suicide attempt (%)		10 (22)	
ICV, mean (SD)	1443 (153) ml	1401 (146) ml	0.20
TNF-α, mean; pg/mL (SD)	1.270 (0.365)	1.602 (0.607)	<0.01

*SD* standard deviation, *ICV* intracranial volume, *MDD* major depression disorders, *HAMD-17* 17-item Hamilton Rating Scale for Depression, *TNF* tumor necrosis factor.

Thirty-eight right-handed HSs were also recruited from nearby communities via an interview conducted by the same psychiatrist using the full SCID-I/NP. None of the HSs had a history of serious medical or neuropsychiatric illness, or a family history of major psychiatric or neurological illness among their first-degree relatives (Table 1).

A radiologist (S.K., 22 years of experience in neuroradiology) who reviewed the conventional MRI data (including T2-weighted images) reported no gross abnormalities, such as infarcts, hemorrhaging, or brain tumors, in any of the study participants.

### Cytokine analyses

All human blood samples were assayed in singlicate (due to limited sample volumes) using the V-PLEX Human Proinflammatory Panel I (4-Plex), which is a highly sensitive multiplex enzyme-linked immunosorbent assay used to quantitatively measure TNF-α, from a single small sample volume (25 μL) using an electrochemiluminescent detection method (MesoScale Discovery, Gaithersburg, MD, USA). The mean intra-assay coefficients, based on the standards run in duplicate for each cytokine, were <8.5% for all cytokines. For the statistical analysis, any value that was below the lowest limit of detection (LLOD) for the cytokine assay was replaced with half of the LLOD of the assay. This imputation method is robust and well established^15^.

### MRI

MRI was performed using a 3T MR system (Signa EXCITE 3T; GE Healthcare, Wankesha, WI, USA) with an 8-channel brain phased-array coil. Original T_1_ images were acquired by three-dimensional fast-spoiled gradient recalled acquisition in the steady state. The acquisition parameters were as follows: repetition time, 10 ms; echo time, 4.1 ms; inversion time, 700 ms; flip angle, 10; field-of view, 24 cm; section thickness, 1.2 mm; and resolution, 0.9 × 0.9 × 1.2 mm. All images were corrected for image distortion due to gradient non-linearity using the Grad Warp software program^16^ and for intensity inhomogeneity with the “N3” function^17^.

### Image processing for VBM

A fully automatic technique for the computational analysis of differences in regional brain volume throughout the brain was conducted using the SPM12 software program (Statistical Parametric Mapping 12; Institute of Neurology, London, UK)^18,19^. The 3D-FSPGR images in native space were spatially normalized, segmented into GM, white matter, and cerebrospinal fluid images, and modulated using the Diffeomorphic Anatomical Registration Through Exponential Lie Algebra (DARTEL) toolbox in SPM12^20^. The DARTEL was proposed by Ashburner as an alternative method for normalization in the SPM software package^18^. To preserve the gray and white matter volumes within each voxel, we modulated the images using the Jacobean determinants derived from the spatial normalization by DARTEL. The resulting modulated GM images were smoothed using an 8 mm full-width at half-maximum Gaussian kernel.

### Image processing for SBM

SBM is a multivariate technique that takes advantage of independent component analyses (ICAs)^13^. SBM takes into account information across different voxels and identifies unpredicted, naturally occurring patterns of covariance across brain regions. The preprocessing of images is identical to the procedure adopted for classical VBM analyses.

For image processing, a SBM analysis was carried out using the GIFT toolbox (http://icatb.sourceforge.net)^13^. The minimum description length (MDL) principle was used to estimate the number of independent components. The MDL identified 17 reliable independent components (GM structural networks). We performed ICAs using a neural network algorithm (Infomax) that attempted to minimize the mutual information of the network outputs in order to identify naturally grouping and maximally independent sources^21^. The ICAs were repeated 20 times in ICASSO (http://research.ics.aalto.fi/ica/icasso/), and the resulting components were clustered to ensure their consistency and reliability.

Group comparisons between MDD and HCs used mean *Z*-scores based on the same independent component (GM structural network), which were extracted from the SBM analysis. Therefore, according to methods in the previous study^13^, we extracted independent components from the 83 subjects (38 HSs and 45 MDD patients). The preprocessed images from these 83 subjects were arrayed into one 83-row subject-by-GM data matrix. This matrix comprised 83 rows (83 subjects), and each column indicated a voxel. The 83-row subject-by-gray matter data matrix was further decomposed into 2 matrices by the ICA. The first matrix was named the “mixing matrix” and comprised one subject per row and an independent component per column. The mixing matrix involved “loading coefficients” demonstrating how each structural component contributed to the 83 subjects and thus contained information about the relationship between each subject and each component. The second matrix was named the source matrix and specified the relationship between the ICs and the voxels. For GM volume component visualization, the source matrix was reshaped back into a three-dimensional image, scaled to unit standard deviations (Z maps), and thresholded at *Z* > 2.5.

### Statistical analyses

All statistical analyses were performed using the R software program (R 3.1.0, R Foundation for Statistical Computing, Vienna, Austria). *P* values of <0.05 were considered to indicate statistical significance.

As the age and TNF-α levels exhibited Gaussian distributions, we used independent sample *t*-tests to assess the differences between HSs (controls) and patients with MDD (patients). Fisher’s exact test was used for sex comparisons. In the VBM analysis, statistical analyses were performed using the SPM12 software program. The morphological changes in the GM were assessed according to the diagnosis status using a two-sample *t*-test. Age, sex, and total GM volume were included as covariates of no interest in all analyses as confounding variables. The differences in the GM volume between MDD patients and HSs were assessed at the whole-brain level. This analysis yielded statistical parametric maps (SPMs [t]) based on a voxel-level height threshold of *p* < 0.001. We used cluster-level family wise error (FWE) correction. FWE-corrected *p* values of <0.05 were considered to indicate statistical significance.

In the analysis of loading coefficients calculated from SBM, we performed the following analyses: (a) a comparison of GM structural networks between MDD patients and HSs; and (b) an analysis of the linear correlation between GM structural networks and the TNF-α levels. Each subject has a loading coefficient for each component, such that for each component, the groups were compared with the loading coefficients as inputs. The loading coefficients were transformed to *Z*-scores using Fisher’s z-transformation. The *Z*-scores in SBM allow for the identification of sources that exhibit group differences (MDD patients vs. HSs) or particular relationships with other variables of interest (e.g., the TNF-α level). To compare the differences in GM structural networks, we compared the loading coefficients (*Z*-scores) in each component using a two-sample *t*-test. Spearman’s rank correlation was applied to identify the association between serum TNF-α levels and the loading coefficients (*Z*-scores) from each subject. Bonferroni correction for multiple comparisons was applied to the tests by multiplying *P* values by the number of tests, subject to a maximum of 1.0. All results were thresholded at *p* < 0.05 with Bonferroni correction. Thus, *P* values of <0.05 were assumed to indicate a statistically significant difference in all analyses except for the SPM12 analysis.

## Results

### Demographic data

The Table 1 shows the participants’ demographic data. There were no significant differences in the age, gender, or intracranial volume on MRI between HSs and patients. The serum TNF-α levels of the patients were significantly higher than in HSs. The serum TNF-α level was associated with the total HAMD-17 score in the MDD patients (*r* = −0.350, *p* = 0.01 by Spearman’s rank correlation).

### VBM analyses

The whole-brain analysis showed no significant differences in the regional GM volume between patients and HSs.

### SBM analyses

The ICA generated 17 independent components (GM structural networks). Three of these components were determined to be artifacts based on the criteria defined by Xu et al. (2009): components containing several sharp edges near the boundary of the brain or appearing primarily in regions that do not contain GM^13^. Previously, Williams reviewed the neural circuit taxonomy regarding depression and anxiety based on published work, which including various cerebral neural circuits, but not cerebellar circuits^22^. Based on that article, four independent components, which mainly included cerebellar networks, were excluded from the subsequent analysis. Thus, from among 14 independent components, we extracted a total of 10 independent components.

**Fig. 1:**
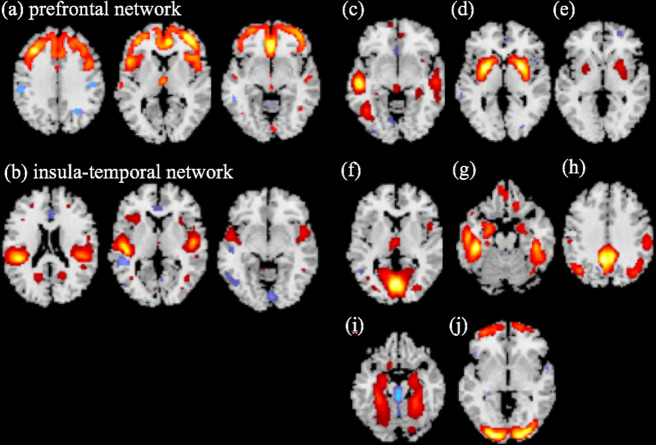
Sources discovered by SBM. Source-based morphometry revealed 10 structural networks (**a**–**j**). Regarding (**a**) the prefrontal network and (**b**) insula-temporal network, the mean *Z-*scores of MDD patients were significantly lower than those of HCs after Bonferroni correction (−0.305 ± 0.85 and 0.253 ± 0.82; *P* = 0.03 in the prefrontal network, and −0.268 ± 0.86 and 0.467 ± 0.71; *P* < 0.01 in the insula-temporal network). The other structural networks (**c**–**j**), which are generated from SBM analyses, showed no significant differences between MDD patients and HSs.

Of these 10 independent components, there were only 2 for which significant differences were observed between MDD patients and HSs (*P* < 0.05, with Bonferroni correction applied by multiplying *P* values by the number of tests [*n* = 10]). We called these MDD-specific components “the prefrontal network” and “the insula-temporal network” (Fig. 1). For the two components, the mean *Z*-scores in MDD patients were significantly lower than those in HCs after Bonferroni correction: (−0.305 ± 0.85 and 0.253 ± 0.82; nominal *P* < 0.01 and corrected *P* with Bonferroni correction = 0.03 in the prefrontal network) and (−0.268 ± 0.86 and 0.467 ± 0.71; nominal *P* < 0.01 and corrected *P* with Bonferroni correction <0.01 in the insula-temporal network).

**Fig. 2:**
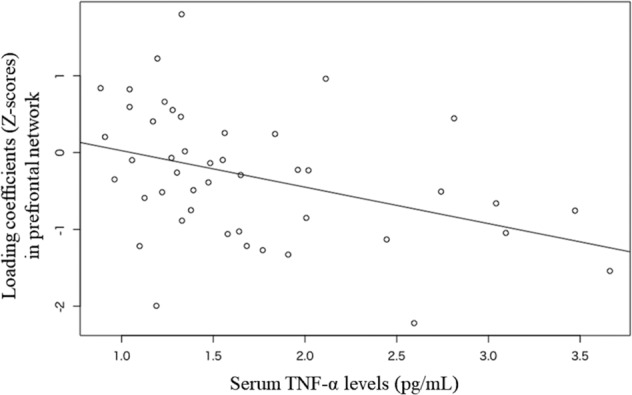
Loading coefficients (*Z*-scores) in the prefrontal network and serum TNF-α levels in MDD patients. The serum TNF-α levels are significantly correlated with the *Z*-scores in the prefrontal network after Bonferroni correction (*r* = −0.419, *p* < 0.01).

To identify whether or not serum TNF-α affected the two GM structural networks (the prefrontal and insula-temporal networks), we assessed the correlation between the *Z*-scores and the serum TNF-α levels. The serum TNF-α levels were significantly correlated with the *Z*-score of prefrontal network after Bonferroni correction was applied by multiplying *P* values by the number of tests [*n* = 2] (*r* = −0.419, nominal *P* < 0.01 and corrected *P* with Bonferroni correction <0.01) (Fig. 2); however, the correlation was not significant for the insula-temporal network (*r* = −0.290, *p* = 0.11).

There was no significant linear correlation between the *Z*-scores and total HAM-D score in the two components (the prefrontal network and the insula-temporal network).

## Discussion

In this study, the *Z*-scores of MDD patients in the prefrontal network were significantly lower than those of HSs, which may indicate the presence of abnormal GM structural networks in patients with MDD. Furthermore, in the MDD patients, the *Z*-scores in the prefrontal network showed significant negative correlations with the serum TNF-α levels. Thus, our results suggest that neuroinflammation (indicated by an elevated serum TNF-α level) in the early stage of MDD is associated with prefrontal network alterations. Inflammation (indicated by an elevated serum TNF-α level) is considered to be a characteristic of the pathophysiology of MDD^7,8^. We also found that the serum TNF-α levels of MDD patients were significantly higher than those of HSs. Many previous studies have shown that elevated serum TNF-α levels can have detrimental effects on the central nervous system^23–25^. Yang et al.^26^ showed that serum TNF-α could affect the brain structure via dendritic elimination, independent of central inflammatory activity. In addition, some previous studies using a preclinical mouse model revealed that serum TNF-α is critical to the development of various cortices^27,28^. These studies support our results.

Segall et al.^29^ investigated the correspondence between GM structural networks using SBM and functional networks using resting-state fMRI and identified several structural components that corresponded to resting-state functional components. Alexander-Bloch et al.^30^ also showed that the functional network identified using resting-state fMRI was somewhat predictable by GM structural networks. Scheinost et al. also assessed covariances in the regional brain structure and function applied to structural and functional MRI data in unmedicated MDD patients and found a higher structure-to-function correlation in PFC in the MDD group than the HSs^31^. In addition, both analyses revealed alterations in multiple brain networks centered around the PFC in MDD patients compared with HSs. These results support our present findings, in which the *Z*-scores of MDD patients in the prefrontal network were significantly lower than those of HSs.

A meta-analysis on depression and anxiety showed connectivity dysfunction within and between various brain circuits, including the positive-affect circuit, the default mode circuit, the attention circuit, and the cognitive control circuit^22^. The PFC is considered to play key roles in these circuits. The positive-affect circuit, which is the reward-processing components of the affective circuits, is defined by the striatal nucleus accumbens and ventral tegmental areas and their projections into the orbitofrontal cortex and medial PFC^32^. Many previous studies have suggested the presence of dysfunctional positive-affect processing under conditions of depression and anxiety^33–35^. The default mode circuit is defined by the anterior medial PFC, the posterior cingulate cortex, and the angular gyrus^36^. In a previous study, default-mode hypoconnectivity was correlated with social anxiety^37^. The frontoparietal attention circuit is defined by nodes in the medial superior PFC, anterior insula, anterior inferior parietal lobule, and precuneus^38^. Dysfunction of the frontoparietal attention circuit has also been observed in cases of social anxiety^39^. The cognitive control circuit is composed of the dorsolateral PFC, anterior cingulate cortex, dorsal parietal cortex, and precentral gyrus. Notably, task-evoked dorsolateral PFC dysfunction in the cognitive control circuit has been observed in unmedicated MDD patients^40^.

In the current study, the voxel-based analysis demonstrated no significant differences in the regional GM volume between MDD patients and HSs. This may indicate that the brain changes in the early stage of first-episode MDD may be too subtle to be locally detectable by VBM. However, previous studies using other imaging techniques, such as surface-based morphometry^41–43^ and functional MRI^31^, detected brain alterations in several regions in the early stage of first-episode MDD. Thus, in addition to surface-based morphometry and functional MRI, SBM may also be suitable for studying early stage MDD, since anatomical changes are likely to be distributed along networks of brain regions.

Network science has provided powerful analytical tools for examining the complex interactions of cerebral organization. Several different methods for constructing morphological brain networks have been established^44–46^. In these studies, brain networks were constructed by segmenting the brain into discrete regions (denoted as nodes) based on the gyral-based anatomical atlas, such as the Desikan–Killiany Atlas^47^, and then the coupling relationship between two nodes (denoted as the edge) was determined. However, the relationship between the morphological anatomy based on the gyral-based anatomical atlas and actual functional anatomy is unclear. Our SBM is a multivariate technique that takes advantage of ICAs. The SBM takes into account information across different voxels and identifies unpredicted, naturally occurring patterns of covariance across brain regions. This data-driven, voxel-based method, which examines the intrinsic connectivity of each voxel to every other voxel in the brain, does not require prior knowledge for selecting regions or networks of interest, such as a gyral-based anatomical atlas. Therefore, we believe that the SBM based on ICAs is more useful for understanding the actual functional connectivity in the human brain.

This study was limited by the small sample size, as SBM is expected to show better performance with more data^13^. For instance, the number of components that were able to be extracted was proportional to the number of participants^48^. We therefore tried to include more participants in our study. However, it was difficult to recruit and retain drug-naïve patients with MDD during their first episode, as many patients received antidepressants before they underwent MRI. We focused on the cross-sectional association based on one-time assessments of inflammatory marker levels; however, these measurements cannot reliably distinguish between chronic and acute inflammation. The few previous studies that have assessed chronic inflammation have revealed stronger associations with mental health when inflammation is determined using repeated measurements rather than a single measurement^49^.

In conclusion, the serum TNF-α levels in patients with MDD were significantly higher than in HSs. Importantly, the elevated serum TNF-α levels in the early stage of MDD were associated with GM structural network alterations in the PFC. This result supports the findings of a previous animal study, suggesting that TNF-α-induced neuronal changes in the PFC have a pathophysiological role in MDD^10^. The use of SBM based on ICAs allowed us to identify the various brain networks that showed significant differences between MDD patients and HSs. SBM may contribute to neural circuit taxonomy for depression and anxiety.
